# Crystal structure of (*Z*)-2-(5-fluoro-2-oxoindolin-3-yl­idene)hydrazinecarbo­thio­amide

**DOI:** 10.1107/S2056989015008609

**Published:** 2015-05-09

**Authors:** Viviane C. D. Bittencourt, Vitor Y. G. Almeida, Davi F. Back, Vanessa C. Gervini, Adriano Bof de Oliveira

**Affiliations:** a Escola de Química e Alimentos, Universidade Federal do Rio Grande, Av. Itália km 08, Campus Carreiros, 96203-900 Rio Grande-RS, Brazil; b Departamento de Química, Universidade Federal de Santa Maria, Av. Roraima s/n, Campus Universitário, 97105-900 Santa Maria-RS, Brazil; c Departamento de Química, Universidade Federal de Sergipe, Av. Marechal Rondon s/n, Campus Universitário, 49100-000 São Cristóvão-SE, Brazil

**Keywords:** crystal structure, thio­semicarbazone derivative, isatin, two-dimensional hydrogen-bonding network, natural product

## Abstract

In the title compound, C_9_H_7_FN_4_OS, the mol­ecules are almost planar, with an r.m.s. deviation of 0.047 (3) Å from the mean plane defined by the non-H atoms and a maximum deviation of 0.123 (2) Å for the amine N atom. The torsion angle for the N—N—C—S unit is 176.57 (19)°. In the crystal, mol­ecules are linked into inversion dimers *via* pairs of N—H⋯F hydrogen bonds and, additionally, through N—H⋯O and N—H⋯S hydrogen bonds, building a two-dimensional hydrogen-bond network parallel to the (103) plane. An intra­molecular N—H⋯O inter­action is also observed.

## Related literature   

For one of the first reports of the synthesis of thio­semicarbazone derivatives, see: Freund & Schander (1902 ▸). For the synthesis and crystal structure of a similar compound, namely (*Z*)-2-(5-fluoro-2-oxoindolin-3-yl­idene)-*N*-phenyl­hydrazinecarbo­thio­amide, see: Ali *et al.* (2012 ▸). For a review on hydrogen bonding, see: Steiner (2002 ▸).
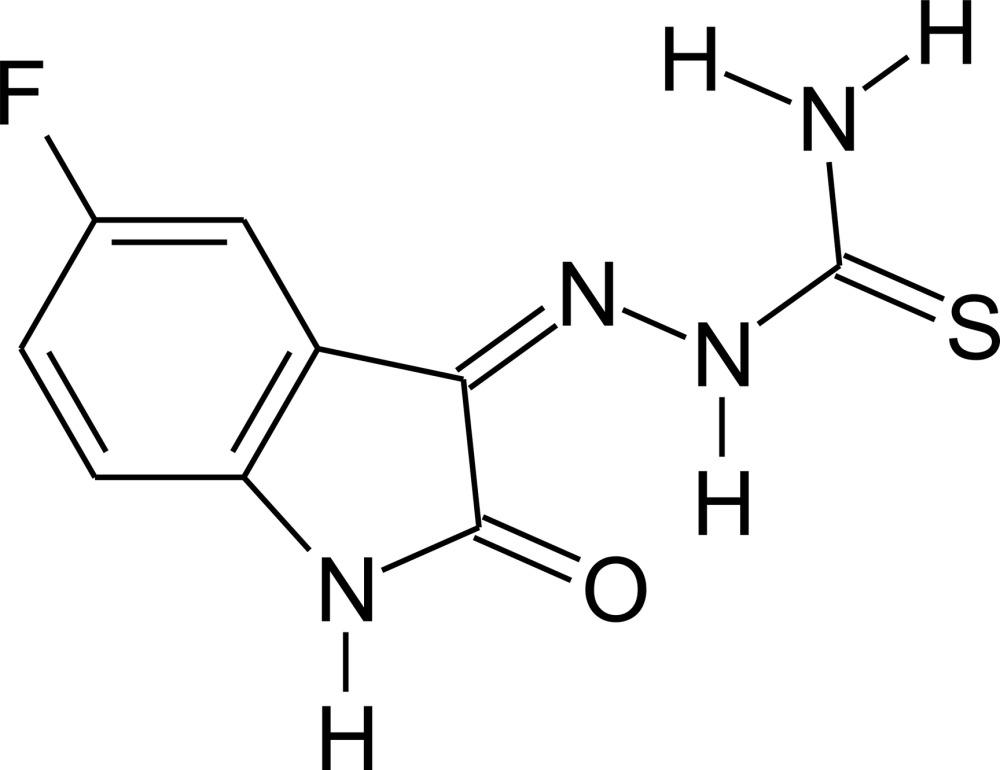


## Experimental   

### Crystal data  

C_9_H_7_FN_4_OS
*M* *_r_* = 238.25
Monoclinic, 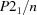

*a* = 4.7151 (1) Å
*b* = 15.4517 (4) Å
*c* = 13.9645 (4) Å
β = 93.921 (2)°
*V* = 1015.02 (4) Å^3^
*Z* = 4
Mo *K*α radiation
μ = 0.32 mm^−1^
*T* = 293 K
0.44 × 0.16 × 0.11 mm

### Data collection  

Bruker X8 Kappa APEXII diffractometer
Absorption correction: numerical (*SADABS*; Bruker 2009 ▸) *T*_min_ = 0.954, *T*_max_ = 0.966
12531 measured reflections
2239 independent reflections
1390 reflections with *I* > 2σ(*I*)
*R*_int_ = 0.064

### Refinement  

*R*[*F* ^2^ > 2σ(*F* ^2^)] = 0.049
*wR*(*F* ^2^) = 0.140
*S* = 1.01
2239 reflections
145 parameters
H-atom parameters constrained
Δρ_max_ = 0.20 e Å^−3^
Δρ_min_ = −0.29 e Å^−3^

### 

Data collection: *APEX2* (Bruker, 2009 ▸); cell refinement: *SAINT* (Bruker, 2009 ▸); data reduction: *SAINT*; program(s) used to solve structure: *SHELXS97* (Sheldrick, 2008 ▸); program(s) used to refine structure: *SHELXL97* (Sheldrick, 2008 ▸); molecular graphics: *DIAMOND* (Brandenburg, 2006 ▸); software used to prepare material for publication: *publCIF* (Westrip, 2010 ▸).

## Supplementary Material

Crystal structure: contains datablock(s) I, publication_text. DOI: 10.1107/S2056989015008609/lr2136sup1.cif

Structure factors: contains datablock(s) I. DOI: 10.1107/S2056989015008609/lr2136Isup2.hkl

Supporting information file. DOI: 10.1107/S2056989015008609/lr2136Isup3.cml

**Fig. 1.**
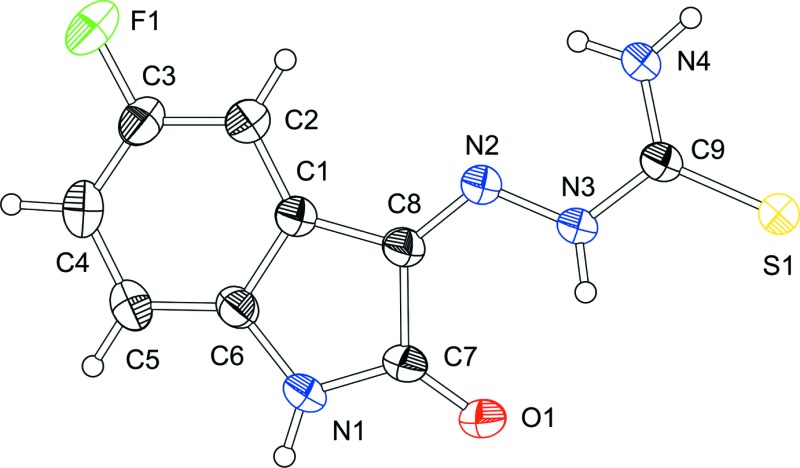
: The molecular structure of the title compound with labeling and displacement ellipsoids drawn at the 40% probability level. H atoms are drawn isotropically.

**Fig. 2.**
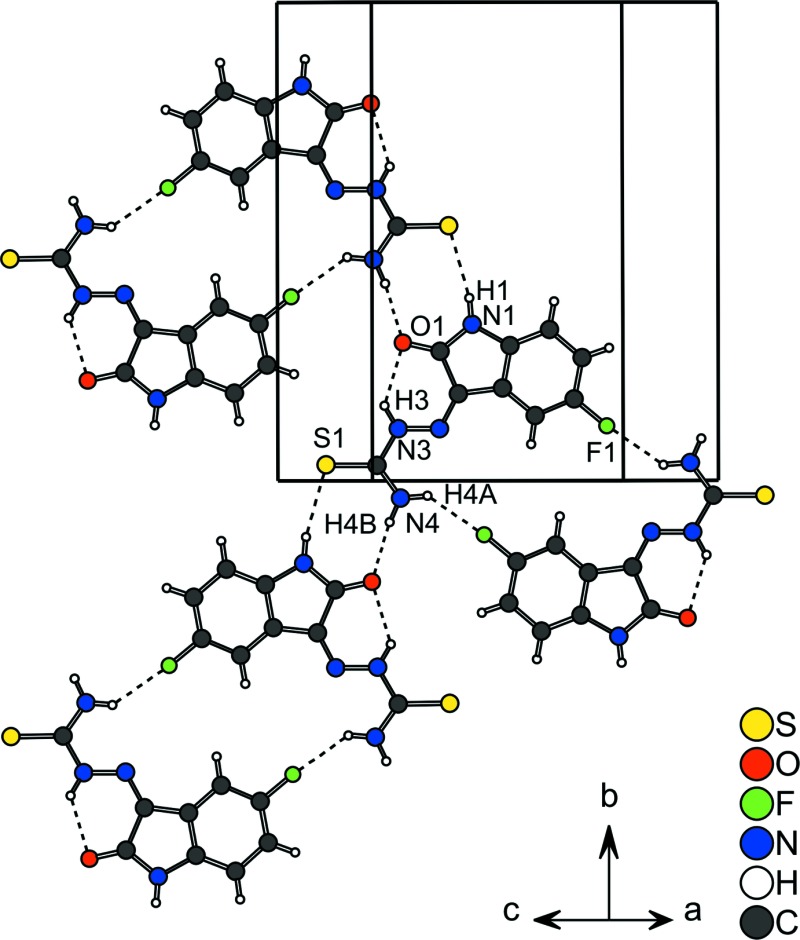
: A view, down the c axis, of the packing of the title compound showing the two dimensional hydrogen-bond network. Hydrogen bonds are shown as dashed lines.

. DOI: 10.1107/S2056989015008609/lr2136fig1.tif
The mol­ecular structure of the title compound with labeling and displacement ellipsoids drawn at the 40% probability level. H atoms are drawn isotropically.

c . DOI: 10.1107/S2056989015008609/lr2136fig2.tif
A view, down the *c* axis, of the packing of the title compound showing the two dimensional hydrogen-bond network. Hydrogen bonds are shown as dashed lines.

CCDC reference: 1062930

Additional supporting information:  crystallographic information; 3D view; checkCIF report

## Figures and Tables

**Table 1:** Hydrogen-bond geometry (, )

*D*H*A*	*D*H	H*A*	*D* *A*	*D*H*A*
N3H3O1	0.86	2.12	2.781(3)	133
N1H1S1^i^	0.86	2.55	3.367(2)	158
N4H4*A*F1^ii^	0.86	2.24	2.956(3)	140
N4H4*B*O1^iii^	0.86	2.03	2.879(3)	171

Symmetry codes: (i) 
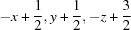
; (ii) 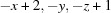; (iii) 
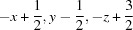
.

## supplementary crystallographic information

### S1. Structural commentary

\ Concerning our inter­est on the study of the supra­molecular chemistry of thio­semicarbazone derivatives from natural products, we report herein the crystal structure of the (*Z*)-2-(5-fluoro-2-oxoindolin-3-yl­idene)-*N*-\ hydrazinecarbo­thio­amide, a thio­semicarbazone derivative from 5-fluorisatin (Fig. 1). The molecular structure of the title compound matches the asymmetric unit and itsn't planar. The maximum deviation from the mean plane of the non-H atoms amounts to 0.1229 (22) Å for N4 and the torsion angle for the N2–N3–C9–S1–entity amounts to 176.57 (19)°. All bond distances and angles are consistent with literature data of a similiar compound, (*Z*)-2-(5-fluoro-2-oxoindolin-3-yl­idene)-*N*-\ phenyl­hydrazinecarbo­thio­amide (Ali *et al.*, 2012). In the crystal of the title compound, the molecules are linked into dimers *via* pairs of N4—H4A···F1 hydrogen bonds. The dimers are linked into a two dimensional hydrogen bonded network through the N1—H1···S1, O1···H4B—N4 and S1···H1—N1 hydrogen bonds. In addition, an intra­molecular N3—H3···O1 inter­action is also observed and the O1 atom builds a bifurcated hydrogen bonding with the H3 and H4B atoms (Table 1, Fig. 2 and Steiner, 2002). The 2-D H-bonded polymers are stacked along the *a*-axis without any strong or relevant inter­molecular inter­actions between themselves. The molecules of the title compound are also related by two fold screw axis parallel to the *b*-direction (Fig. 2)

### S2. Synthesis and crystallization

Starting materials were commercially available and were used without further purification. The synthesis was adapted from a procedure reported previously (Freund & Schander, 1902). The hydro­chloric acid catalyzed reaction of 5-fluorisatin (8,83 mmol) and thio­semicarbazide (8,83 mmol) in ethanol (50 ml) was refluxed for 6 h. After cooling and filtering, the title compound was obtained. Crystals suitable for X-ray diffraction of title compound were obtained in ethanol by the slow evaporation of the solvent.

### S3. Refinement

All aromatic H atoms were positioned with idealized geometry and were refined isotropic with *U*_iso_(H) = 1.2 *U*_eq_(C) using a riding model with C—H = 0.93 Å. The other H atoms were located in difference map but were positioned with idealized geometry and refined isotropic with *U*_iso_(H) = 1.2 *U*_eq_(N) using a riding model with N—H = 0.86 Å.

### Crystal data

**Table:**

C_9_H_7_FN_4_OS	*F*(000) = 488
*M**_r_* = 238.25	*D*_x_ = 1.559 Mg m^−^^3^
Monoclinic, *P*2_1_/*n*	Mo *K*α radiation, λ = 0.71073 Å
Hall symbol: -P 2yn	Cell parameters from 1744 reflections
*a* = 4.7151 (1) Å	θ = 2.6–22.4°
*b* = 15.4517 (4) Å	µ = 0.32 mm^−^^1^
*c* = 13.9645 (4) Å	*T* = 293 K
β = 93.921 (2)°	Block, orange
*V* = 1015.02 (4) Å^3^	0.44 × 0.16 × 0.11 mm
*Z* = 4	

### Data collection

**Table:**

Bruker X8 Kappa APEXII diffractometer	2239 independent reflections
Radiation source: fine-focus sealed tube, Bruker X8 Kappa APEX II	1390 reflections with *I* > 2σ(*I*)
Graphite monochromator	*R*_int_ = 0.064
Detector resolution: 8.3333 pixels mm^-1^	θ_max_ = 27.2°, θ_min_ = 2.0°
0.5 ° ω & φ scans	*h* = −3→6
Absorption correction: numerical (*SADABS*; Bruker 2009)	*k* = −19→19
*T*_min_ = 0.954, *T*_max_ = 0.966	*l* = −17→17
12531 measured reflections	

### Refinement

**Table:**

Refinement on *F*^2^	Primary atom site location: structure-invariant direct methods
Least-squares matrix: full	Secondary atom site location: difference Fourier map
*R*[*F*^2^ > 2σ(*F*^2^)] = 0.049	Hydrogen site location: inferred from neighbouring sites
*wR*(*F*^2^) = 0.140	H-atom parameters constrained
*S* = 1.01	*w* = 1/[σ^2^(*F*_o_^2^) + (0.0642*P*)^2^ + 0.1779*P*] where *P* = (*F*_o_^2^ + 2*F*_c_^2^)/3
2239 reflections	(Δ/σ)_max_ < 0.001
145 parameters	Δρ_max_ = 0.20 e Å^−^^3^
0 restraints	Δρ_min_ = −0.29 e Å^−^^3^

### Special details

**Table:**

Geometry. All e.s.d.'s (except the e.s.d. in the dihedral angle between two l.s. planes) are estimated using the full covariance matrix. The cell e.s.d.'s are taken into account individually in the estimation of e.s.d.'s in distances, angles and torsion angles; correlations between e.s.d.'s in cell parameters are only used when they are defined by crystal symmetry. An approximate (isotropic) treatment of cell e.s.d.'s is used for estimating e.s.d.'s involving l.s. planes.
Refinement. Refinement of *F*^2^ against ALL reflections. The weighted *R*-factor *wR* and goodness of fit *S* are based on *F*^2^, conventional *R*-factors *R* are based on *F*, with *F* set to zero for negative *F*^2^. The threshold expression of *F*^2^ > σ(*F*^2^) is used only for calculating *R*-factors(gt) *etc*. and is not relevant to the choice of reflections for refinement. *R*-factors based on *F*^2^ are statistically about twice as large as those based on *F*, and *R*- factors based on ALL data will be even larger.

### Fractional atomic coordinates and isotropic or equivalent isotropic displacement parameters (Å^2^)

**Table:**

	*x*	*y*	*z*	*U*_iso_*/*U*_eq_	
S1	0.02917 (16)	0.03364 (5)	0.86791 (6)	0.0558 (3)	
O1	0.3669 (4)	0.28879 (12)	0.73620 (14)	0.0506 (5)	
F1	1.2874 (4)	0.11434 (14)	0.40106 (15)	0.0840 (7)	
N3	0.3467 (4)	0.10914 (14)	0.74614 (16)	0.0452 (6)	
H3	0.2958	0.1572	0.7708	0.054*	
N1	0.6921 (4)	0.32661 (14)	0.62633 (16)	0.0472 (6)	
H1	0.6864	0.3820	0.6319	0.057*	
N2	0.5310 (4)	0.10861 (14)	0.67623 (16)	0.0433 (5)	
N4	0.3217 (5)	−0.03703 (14)	0.73181 (19)	0.0587 (7)	
H4A	0.4327	−0.0322	0.6857	0.070*	
H4B	0.2628	−0.0872	0.7481	0.070*	
C1	0.8212 (5)	0.19327 (16)	0.57361 (19)	0.0411 (6)	
C8	0.6233 (5)	0.18194 (16)	0.64722 (19)	0.0402 (6)	
C7	0.5406 (5)	0.27122 (16)	0.67762 (19)	0.0413 (6)	
C6	0.8603 (5)	0.28250 (17)	0.56272 (19)	0.0412 (6)	
C5	1.0436 (6)	0.31552 (19)	0.4996 (2)	0.0502 (7)	
H5	1.0706	0.3748	0.4936	0.060*	
C3	1.1423 (6)	0.1702 (2)	0.4555 (2)	0.0549 (8)	
C2	0.9634 (5)	0.13570 (19)	0.5189 (2)	0.0507 (7)	
H2	0.9393	0.0762	0.5246	0.061*	
C9	0.2420 (5)	0.03249 (16)	0.7774 (2)	0.0431 (6)	
C4	1.1869 (6)	0.2569 (2)	0.4450 (2)	0.0553 (8)	
H4	1.3129	0.2766	0.4015	0.066*	

### Atomic displacement parameters (Å^2^)

**Table:**

	*U*^11^	*U*^22^	*U*^33^	*U*^12^	*U*^13^	*U*^23^
S1	0.0683 (5)	0.0365 (4)	0.0666 (5)	−0.0055 (3)	0.0331 (4)	−0.0047 (3)
O1	0.0629 (11)	0.0349 (10)	0.0560 (12)	0.0082 (9)	0.0181 (10)	−0.0011 (9)
F1	0.0816 (12)	0.0853 (15)	0.0906 (15)	0.0084 (11)	0.0470 (11)	−0.0180 (12)
N3	0.0534 (12)	0.0273 (11)	0.0571 (15)	0.0001 (9)	0.0209 (11)	−0.0011 (10)
N1	0.0558 (13)	0.0259 (11)	0.0614 (15)	−0.0024 (9)	0.0153 (12)	0.0024 (10)
N2	0.0457 (11)	0.0331 (12)	0.0526 (14)	0.0004 (9)	0.0150 (10)	−0.0001 (10)
N4	0.0721 (15)	0.0292 (12)	0.0798 (18)	−0.0060 (11)	0.0412 (14)	−0.0022 (12)
C1	0.0408 (13)	0.0347 (14)	0.0484 (16)	0.0003 (11)	0.0082 (12)	−0.0001 (11)
C8	0.0407 (13)	0.0317 (14)	0.0486 (16)	0.0020 (11)	0.0064 (12)	0.0014 (11)
C7	0.0489 (14)	0.0289 (13)	0.0466 (15)	0.0025 (11)	0.0062 (12)	0.0000 (11)
C6	0.0422 (13)	0.0339 (14)	0.0478 (16)	−0.0016 (11)	0.0037 (12)	0.0036 (11)
C5	0.0495 (15)	0.0470 (17)	0.0544 (18)	−0.0081 (13)	0.0065 (13)	0.0096 (14)
C3	0.0513 (16)	0.060 (2)	0.0551 (19)	0.0032 (14)	0.0170 (14)	−0.0062 (15)
C2	0.0497 (14)	0.0437 (16)	0.0604 (19)	0.0022 (13)	0.0157 (13)	−0.0020 (14)
C9	0.0452 (14)	0.0314 (13)	0.0537 (17)	−0.0003 (11)	0.0106 (12)	0.0004 (12)
C4	0.0484 (15)	0.068 (2)	0.0514 (18)	−0.0079 (14)	0.0127 (14)	0.0075 (15)

### Geometric parameters (Å, º)

**Table:**

S1—C9	1.667 (3)	C1—C2	1.377 (4)
O1—C7	1.227 (3)	C1—C6	1.401 (4)
F1—C3	1.365 (3)	C1—C8	1.446 (3)
N3—N2	1.351 (3)	C8—C7	1.503 (3)
N3—C9	1.366 (3)	C7—O1	1.227 (3)
N3—H3	0.8600	C6—C5	1.374 (3)
N1—C7	1.351 (3)	C5—C4	1.388 (4)
N1—C6	1.407 (3)	C5—H5	0.9300
N1—H1	0.8600	C3—C4	1.365 (4)
N2—C8	1.289 (3)	C3—C2	1.371 (4)
N4—C9	1.316 (3)	C2—H2	0.9300
N4—H4A	0.8600	C4—H4	0.9300
N4—H4B	0.8600		
			
N2—N3—C9	119.3 (2)	C5—C6—C1	121.9 (2)
N2—N3—H3	120.3	C5—C6—N1	129.2 (3)
C9—N3—H3	120.3	C1—C6—N1	108.9 (2)
C7—N1—C6	111.7 (2)	C6—C5—C4	117.4 (3)
C7—N1—H1	124.2	C6—C5—H5	121.3
C6—N1—H1	124.2	C4—C5—H5	121.3
C8—N2—N3	118.0 (2)	F1—C3—C4	118.3 (3)
C9—N4—H4A	120.0	F1—C3—C2	117.9 (3)
C9—N4—H4B	120.0	C4—C3—C2	123.8 (3)
H4A—N4—H4B	120.0	C3—C2—C1	116.8 (3)
C2—C1—C6	120.2 (2)	C3—C2—H2	121.6
C2—C1—C8	132.8 (2)	C1—C2—H2	121.6
C6—C1—C8	107.0 (2)	N4—C9—N3	115.6 (2)
N2—C8—C1	125.3 (2)	N4—C9—S1	125.6 (2)
N2—C8—C7	128.2 (2)	N3—C9—S1	118.83 (19)
C1—C8—C7	106.4 (2)	C3—C4—C5	119.8 (3)
O1—C7—N1	127.9 (2)	C3—C4—H4	120.1
O1—C7—C8	126.1 (2)	C5—C4—H4	120.1
N1—C7—C8	106.0 (2)		
			
C9—N3—N2—C8	179.0 (2)	C2—C1—C6—N1	179.9 (2)
N3—N2—C8—C1	179.8 (2)	C8—C1—C6—N1	−0.5 (3)
N3—N2—C8—C7	−3.6 (4)	C7—N1—C6—C5	−179.5 (3)
C2—C1—C8—N2	−1.7 (5)	C7—N1—C6—C1	−1.0 (3)
C6—C1—C8—N2	178.8 (3)	C1—C6—C5—C4	1.1 (4)
C2—C1—C8—C7	−178.9 (3)	N1—C6—C5—C4	179.5 (3)
C6—C1—C8—C7	1.5 (3)	F1—C3—C2—C1	179.4 (3)
C6—N1—C7—O1	−177.1 (3)	C4—C3—C2—C1	0.6 (5)
C6—N1—C7—C8	1.9 (3)	C6—C1—C2—C3	0.5 (4)
N2—C8—C7—O1	−0.2 (5)	C8—C1—C2—C3	−178.9 (3)
C1—C8—C7—O1	176.9 (3)	N2—N3—C9—N4	−3.3 (4)
N2—C8—C7—N1	−179.2 (3)	N2—N3—C9—S1	176.57 (19)
C1—C8—C7—N1	−2.1 (3)	F1—C3—C4—C5	−179.8 (3)
C2—C1—C6—C5	−1.4 (4)	C2—C3—C4—C5	−0.9 (5)
C8—C1—C6—C5	178.2 (2)	C6—C5—C4—C3	0.0 (4)

### Hydrogen-bond geometry (Å, º)

**Table:**

*D*—H···*A*	*D*—H	H···*A*	*D*···*A*	*D*—H···*A*
N3—H3···O1	0.86	2.12	2.781 (3)	133
N1—H1···S1^i^	0.86	2.55	3.367 (2)	158
N4—H4*A*···F1^ii^	0.86	2.24	2.956 (3)	140
N4—H4*B*···O1^iii^	0.86	2.03	2.879 (3)	171

Symmetry codes: (i) −*x*+1/2, *y*+1/2, −*z*+3/2; (ii) −*x*+2, −*y*, −*z*+1; (iii) −*x*+1/2, *y*−1/2, −*z*+3/2.
