# Supplementary figures and images for: Crystal structure of (Z)-2-(5-fluoro-2-oxoindolin-3-yl­idene)hydrazinecarbo­thio­amide
Source: Acta Crystallogr E Crystallogr Commun. 2015 May 9;71(Pt 6):o383–4. doi: 10.1107/S2056989015008609 (PMC4459295; doi:10.1107/S2056989015008609)

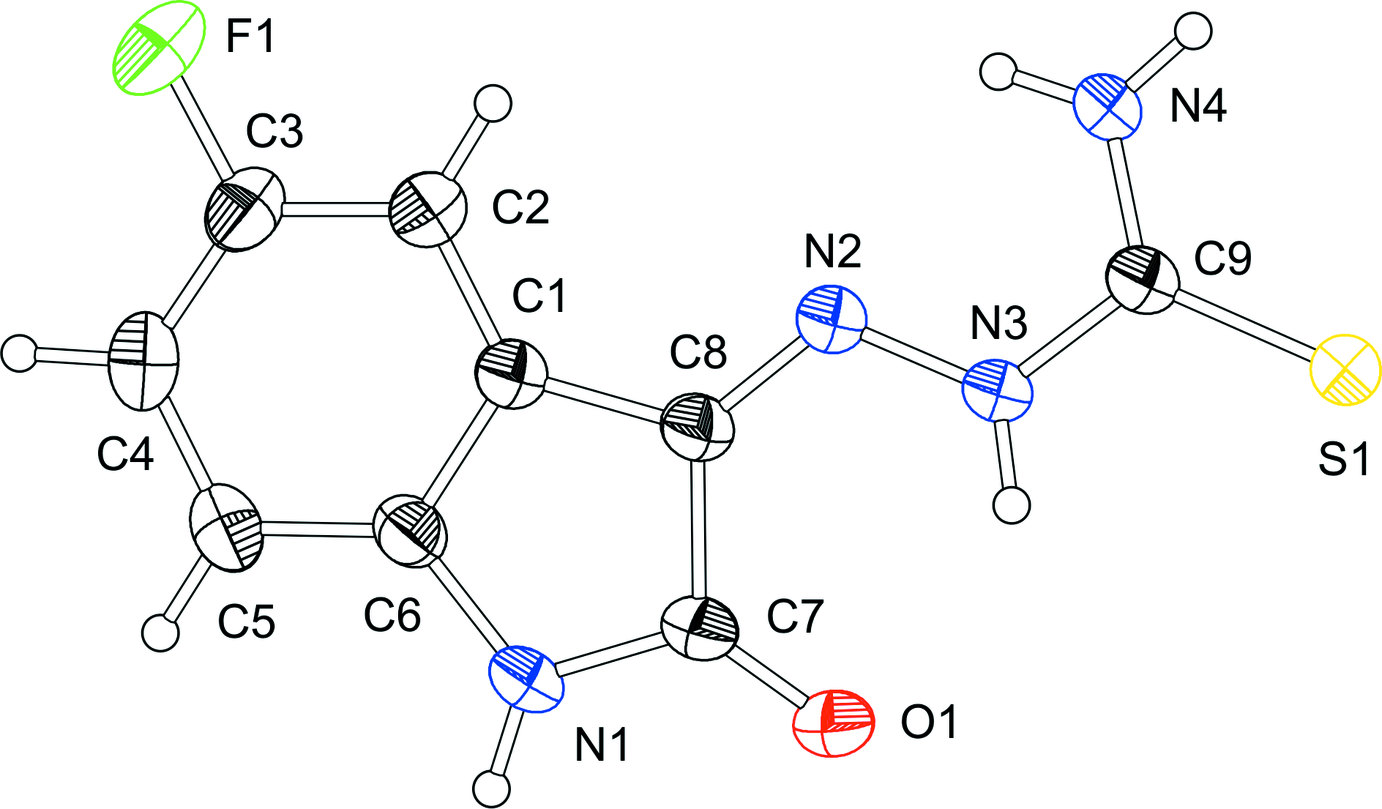

Supplement: Supplementary file 4 [file e-71-0o383-fig1.tif]

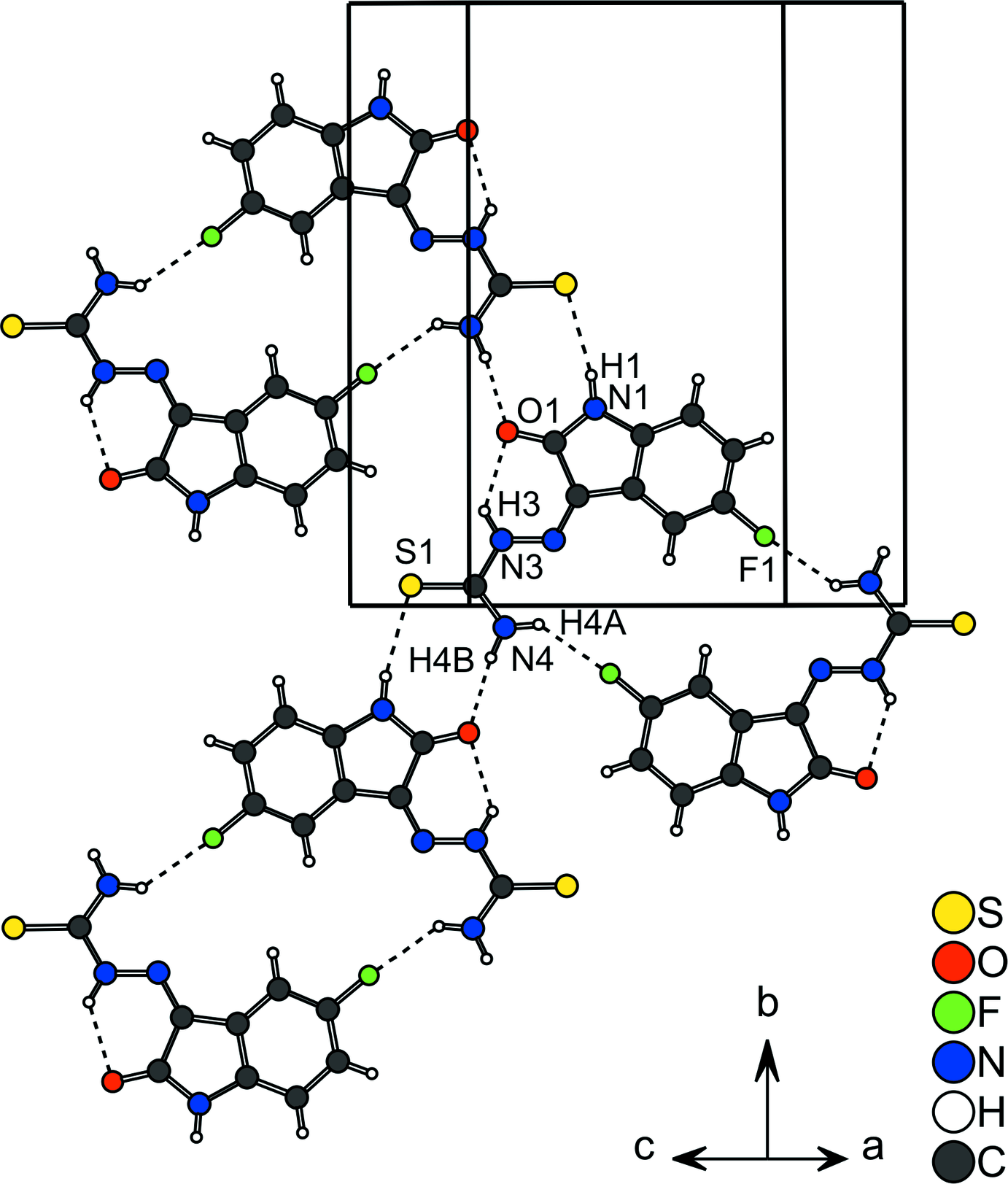

Supplement: Supplementary file 5 [file e-71-0o383-fig2.tif]
